# Performance of a pharmaceutical services regionalization strategy policy in Minas Gerais, Brazil: Pre-post analysis from ERAF project

**DOI:** 10.3389/fphar.2022.953990

**Published:** 2022-09-02

**Authors:** Tatiana Chama Borges Luz, Ana Karine Sarvel de Castro, Isabela Cristina Marques, Betania Barros Cota, Jèssica de Castro Alves, Michael Robert Law

**Affiliations:** ^1^ GETESA (Grupo de Estudos Transdisciplinares em Tecnologias em Saúde e Ambiente), René Rachou Institute (IRR), Oswaldo Cruz Foundation (FIOCRUZ), Belo Horizonte, Brazil; ^2^ Strathclyde Institute of Pharmacy and Biomedical Sciences (SIPBS), Strathclyde University, Glasgow, United Kingdom; ^3^ Centre for Health Services and Policy Research, School of Population and Public Health, The University of British Columbia, Vancouver, CO, Canada

**Keywords:** pre-post study, pharmaceutical policy, medicines, procurement, decomposition analysis

## Abstract

**Background:** In 2016, the Brazilian state of Minas Gerais (∼20 million people), implemented the ERAF policy (“Regionalization Strategy of Pharmaceutical Services”) in an effort to improve medicine procurement and distribution within primary care. We evaluated the impact of the policy on three main goals: price reductions, volume increases, and expansion of therapeutic options.

**Methods:** We analyzed the procurement data from the Integrated System of Management of Pharmaceutical Services database in 2012 and 2018. We estimated the volume, drug mix, and expenditure indicators for all major therapeutic classes, and, in detail, for cardiovascular and nervous system drugs. We evaluated the expenditure drivers using decomposition analyses.

**Results:** Overall, the expenditure increased by 14.5%, drug mix almost doubled, while the volume decreased by a third. Cardiovascular and neurological system drugs followed similar patterns. Decomposition analyses showed that prices and drug mix had positive effects while the volume had negative effects, resulting in an overall increase in expenditure.

**Conclusion:** Our findings suggest that the ERAF policy cannot be considered effective as it has not fulfilled its intended purposes so far. Strategies to address the identified problems and to build a platform for a more sustainable long-lasting policy should be put in place by the government.

## Introduction

The Brazilian National Health Care System (SUS), established more than 30 years ago, covers around 75% of the population for health care services (160 million people) (Brazil, 1998; Andrade et al., 2018). The Pharmaceutical Services based on Primary Care (PHCPS) is a program, part of SUS, funded jointly by the federal, state, and municipal governments. This program was designed to guarantee free access to medicines for patients treated at primary care facilities in Brazil (Costa et al., 2017).

Following the decentralization of health care coverage under SUS, PHCPS is coordinated by municipal governments which are responsible for program management, procurement, distribution of medicines, and provision of care (Brazil, 2017a). Over the past decade, substantial investments in medicine funding have been made in Brazil. The federal government contributed BRL 9.5 billion (∼USD 2.9 billion) toward the program between 2010 and 2016 (Vieira, 2018). It is estimated, however, that the growth in expenditures is more pronounced for the municipalities. For example, Cunha (2014) showed an increase in spending of approximately 230% between 2009 and 2012, with the expenditure increasing from BRL 660 million (∼USD 379 million) to BRL 2.2 billion (∼USD 1.1 billion).

Regardless of these significant financial investments in the PHCPS program, challenges remain with respect to access of medicines for PHCPS patients. Prior studies have suggested deficiencies in the program planning, organization, infrastructure, human resources, and procurement system (Paniz et al., 2016; Luz et al., 2017a, 2017b; Carvalho et al., 2017; Gerlack et al., 2017; Leite et al., 2017; Barbi et al., 2019; Mattos et al., 2019; Soares et al., 2019; Bueno et al., 2021). Problematic sale prices and the failure of suppliers to comply with deadlines and legal documentation requirements have been widespread (CONASS - Conselho Nacional de Secretários de Saúde, 2014; Brazil, 2018a). This has, at least in part, contributed to the resulting shortages of medicines for the population (Helena et al., 2015; Nascimento et al., 2017; Bueno et al., 2021).

In response to these concerns, the state of Minas Gerais implemented a policy known as the Regionalization Strategy of Pharmaceutical Services in Minas Gerais—ERAF (*Estratégia de Regionalização da Assistência Farmacêutica*). This 2016 policy aims to promote technical cooperation between the State and municipal governments to improve medicine procurement and distribution within the PHCPS program. The policy rationale is to promote the purchasing of high-quality products with reliable suppliers obtaining economies of scale at the lowest-possible prices and transaction costs, through a competitive bidding process (SES/MG - Secretaria de Estado de Saúde de Minas Gerais, 2016; Souza Filho et al., 2016). Therefore, under ERAF, the state-level purchasing body is responsible for overarching functions, such as aggregating medication demand from the municipalities, coordinating tenderers eligibility, establishing the contractual instrument (price registration minutes/ata de registro de preços) to record prices, suppliers, and conditions to be applied, and the procurement time frame. Each municipality independently runs their procurement processes and takes buying decisions according to their individual plan and budget, benefiting from the conditions established in the State price registration minute (SES/MG—Secretaria de Estado de Saúde de Minas Gerais, 2016; Souza Filho et al., 2016).

Despite the fact that almost all the municipalities in Minas Gerais joined the ERAF policy (851/853 or 99%), to date, there has not been an evaluation of its impact on the major goals. Therefore, we examined whether the main three goals of the ERAF policy were achieved: reductions in prices, increases in volumes, and an expansion of therapeutic options that were accessed through the PCHPS program.

## Methods

### Research context and study area

This study is part of the ERAF Project, which is part of a mixed-methods study designed to conduct a retrospective, situational, and prospective evaluation of the ERAF Policy in Minas Gerais. This project included both primary data collection and secondary database analyses and qualitative and quantitative methods. The study focused on the State of Minas Gerais, located in the Southeast, Brazil. Within the country, this state has the highest number of municipalities (*n* = 853), the second highest population (20,997,560), and the third largest economy as measured by the gross domestic product (IBGE - Instituto Brasileiro de Geografia e Estatística, 2021). Around two-thirds of the disease burden in Minas Gerais is non-communicable diseases, followed by infectious and parasitic diseases, road injuries, and interpersonal violence (DATASUS - Departamento de Informática do SUS, 2021).

### Data source

This study is based on data extracted from the Integrated System of Management of Pharmaceutical Services (*Sistema Integrado de Gerenciamento da Assistência Farmacêutica*—SIGAF) database. SIGAF is a health information system designed to collect, store, and manage pharmacoepidemiologic and pharmacoeconomic data from the municipalities in the State of Minas Gerais. SIGAF was established as the standard system for planning and monitoring medicine quantification and procurement for all the adherent municipalities within the ERAF Policy. The Minas Gerais State Secretary of Health manages the system and data are made available to researchers upon request (SIGAF - Sistema Integrado de Gerenciamento da Assistência Farmacêutica, 2021).

Data regarding medicine procurement in SIGAF from 2012 and 2018 were analyzed. These 2 years were chosen for comparison purposes considering the data availability, accuracy, completeness, and consistency of the database (Elseviers et al., 2016). Registries were extracted on medicine purchases by municipalities, including drug names, dosage form, strength, purchase date, purchase price, and the number of pharmaceutical units (e.g., tablets, capsules, liquid solutions/suspensions, eye/ear drops, inhalers, suppositories, parenteral injections, transdermal patches, etc.).

The World Health Organization’s Anatomical, Therapeutic, and Chemical (ATC) system was used to classify the medicines into therapeutic groups (WHOCC - WHO Collaborating Centre for Drug Statistics Methodology, 2021). The drugs were aggregated at different ATC levels: the main anatomical group (ATC first level), therapeutic subgroup (ATC second level), and chemical substance (ATC fifth level). Data on the purchasing prices and expenditures were adjusted for inflation using the Extended National Consumer Price Index (IPCA) produced by the Brazilian Institute of Geography and Statistics (IBGE) with 31 December, 2018 as the reference date (IBGE - Instituto Brasileiro de Geografia e Estatística, 2021). The IPCA value for a given month was divided by the IPCA value for 31 December 2018, resulting in the deflation factor for that month. The prices paid in purchases in that month were then multiplied by the deflation factor to give the inflation-adjusted price for each entry in the SIGAF database (over 350,000 entries). These prices were then multiplied by the respective purchasing quantities and totalized to provide the inflation-adjusted expenditure for each month. Expenditure and unit prices were measured in US Dollars (USD) (1 USD = 3,874 Brazilian Reais (BRL) on 31 December 2018). Purchases were aggregated by volume (number of pharmaceutical units purchased) and expenditure (number of pharmaceutical units purchased multiplied by unit purchase price) for 2012 and 2018.

### Pre-post analysis

The pre-post analysis was conducted under three premises: 1) The ERAF Policy is a government intervention directed toward the procurement process of medicines (SES/MG - Secretaria de Estado de Saúde de Minas Gerais); 2) the policy goals are decreasing medicines prices while increasing their acquisition volumes and the expansion of the mix of products (SES/MG - Secretaria de Estado de Saúde de Minas Gerais, 2016); and 3) the understanding of the pharmaceutical spending and its drivers must be considered in formulating and reinforcing pharmaceutical policies (Mousnad et al., 2014). Therefore, two types of analyses were performed as described below. First, the analyses considered all the main anatomical groups (ATC first level) that were purchased, the corresponding chemical substance, and the pharmaceutical products, i.e, all the chemical substances in their different dosage forms and strengths. Following that, the two most relevant therapeutic classes in terms of expenditure and volume—cardiovascular and nervous system drugs, respectively–were evaluated. Microsoft Excel 2007 (Microsoft Corp. United States) was used for the statistical analyses.

### 1) Medicines profile

For the analysis of the medicine profile, absolute and relative indicators for both 2012 and 2018 were constructed, based on previous studies and guidelines (WHO, 2009; Elseviers et al., 2016; Alves et al., 2018; Barbi et al., 2019; Soares et al., 2019; Carvalho et al., 2021).

Three types of absolute indicators were computed: 1) drug mix (D), based on the total number of chemical substances and pharmaceutical products purchased; 2) drug volumes (Annual Volume—AV) by the total number of units of the pharmaceutical products purchased in millions; and 3) drug expenditures (Annual Expenditure—AE) based on the total costs of the pharmaceutical products purchased in millions. The correspondent accumulated totals were also estimated.

In addition, percentage variations were estimated for D, AV, and AE using Eq. 1:

$$Variation{\;}_{\left(D,AV\;orAE\right)}=\left[\frac{\text{Σ}2018-\text{Σ}2012}{\text{Σ}2012}\right]\text{∗}100$$ (1)

The distribution of D, AV, and AE by the major classes of medicines was estimated, as well as the percentage of injectable solutions by the pharmaceutical products purchased.

### 2) Decomposition analysis

The Expenditure Variation Index (E) was estimated by using the decomposition analysis, a technique that detects the driving factors of changes in the pharmaceutical expenditures (Alves et al., 2018; Vieira, 2021).

The analysis is a process of breaking down the expenditure variation into its three components: drug price effects (P), volume effects (V), and drug mix effects (or therapeutic choice or residual effects, D) (Gerdtham et al., 1998; Addis and Magrini, 2002; Alves et al., 2018; Vieira, 2021).

The expenditure variation index (E) was calculated using the following equation:

$$E=PxVxD=\frac{\sum P_{1}V_{0}}{\sum P_{0}V_{0}}X\frac{\sum V_{1}}{\sum V_{0}}X\frac{\left(\sum P_{1}V_{1}/{\sum V}_{1}\right)}{\left(\sum P_{1}V_{0}/\sum V_{0}\right)}$$ (2)

In Eq. 2, P1 and P0 represent the average prices, weighted by purchased quantities, for each pharmaceutical product in periods 1 (2018) and 0 (2012). V1 and V0 represent the volumes in periods 1 (2018) and 0 (2012), respectively. Drugs with the same chemical substance but with different dosage forms and strengths were treated as different products.

In this analysis, an expenditure index greater than one means that the expenditures grew, while values equal to or lower than 1 show stability or a decrease in spending, respectively. When analyzing each component (P, V, or D), a similar interpretation can be applied. Therefore, values greater than 1 indicate that the component contributed to the increase in drug spending; values equal to 1 indicate that no impact from the component was observed and values less than 1 indicate that the component contributed to a decrease in drug spending.

### Ethical considerations

Ethical approval for the ERAF Project was granted by the Ethics Committee of the René Rachou Institute/Fiocruz (Reference: 3.746.752).

## Results

Our study dataset included 801 municipalities in Minas Gerais (94% of all municipalities who adopted the ERAF Policy). In 2012, a total of 1.7 billion pharmaceutical units from 13 different anatomical main groups (ATC first level), 38 therapeutic groups (ATC second level), and 92 chemical substances (ATC fifth level) were purchased by these municipalities. In contrast, in 2018, the total was lower as there were 1.2 billion pharmaceutical units purchased. These purchases, however, were from more ATC sub-groups: 13 different anatomical main groups (ATC first level), 51 therapeutic groups (ATC second level), and 138 chemical substances (ATC fifth level).

### Medicines profile

As shown in Table 1, the total inflation-adjusted medicine expenditures were USD 30.4 million (BRL 117.9 million) in 2012 and USD 34.8 million (BRL 135.0 million) in 2018. This represented a 14.5% increase over these 6 years. The drug classes that had the highest increases in expenditure were, blood and blood-forming organs (+97%), nervous system drugs (+38%), and systemic hormonal preparations (+36%).

**TABLE 1:** Major classes of medicines, drug mix, annual volume, and expenditure in 2012 and in 2018 and variations in the period. Minas Gerais, Brazil.

Major classes of medicines^a^	Drug mix								Annual volume (millions)			Annual expenditure USD (millions) ^b^		
	Chemical substance			Pharmaceutical products^c^										
	2012	2018	Variation^d^ (%)	2012		2018		Total variation^d^ (%)	2012	2018	Variation^d^ (%)	2012	2018	Variation^d^ (%)
				**Total**	**%IS** **^e^**	**Total**	**%IS** **^e^**							
A-alimentary tract and metabolism	8	10	25	9	0	26	15	189	237.67	153.67	−35.34	2.64	3.26	23.54
B-blood and blood forming organs	5	9	80	6	0	14	50	133	82.72	78.12	−5.56	0.58	1.15	96.98
C—cardiovascular system	18	24	33	24	0	47	13	96	816.59	543.50	−33.44	8.85	8.90	0.56
D—dermatologicals	2	8	300	2	0	11	0	450	1.72	0.84	−50.99	0.30	0.34	13.37
G—genito-urinary system and sex hormones	6	6	0	6	33	7	0	17	37.44	0.67	−98.21	1.52	0.24	-84.47
H—systemic hormonal preparations, excl. Sex hormones and insulins	3	6	100	6	0	14	28	133	37.05	51.89	40.08	1.29	1.75	35.49
J—anti-infectives for systemic use	12	13	8	18	11	27	22	50	57.59	40.02	−30.52	4.14	5.21	25.85
M—musculo-skeletal system	3	3	0	6	0	6	0	0	37.84	25.18	−33.45	0.96	0.88	−8.51
N—nervous system	19	24	26	32	6	52	27	63	346.11	256.81	−25.80	7.61	10.51	38.13
P—anti-parasitic products, insecticides, and repellents	6	4	−33	10	0	7	0	-30	9.57	5.38	−43.81	0.37	0.36	−2.75
R—respiratory system	5	8	60	9	0	17	12	89	17.97	15.40	−14.27	1.99	2.15	7.68
S - sensory organs	3	6	100	3	0	7	0	133	0.15	0.09	−41.97	0.13	0.06	−54.88
V—various	1	6	500	1	0	8	75	700	0.10	0.23	123.25	0.05	0.05	4.45
**Total**	91	127	40	132	5	243	20	84	1,682.51	1,171.80	-30.35	30.43	34.84	14.50

a ATC, first level.

b 1USD, 3.8742 BRL.

c Pharmaceutical products including all chemical substances in their different dosage forms and strengths.

d Variation = [(∑2018-∑2012)/∑2012]∗100.

e IS: Injectable Solution.

Along with expenditures, the mix of drugs in terms of the chemical substances purchased also increased. As shown in Table 1, there was a 40% increase between 2012 and 2018, and in terms of pharmaceutical products, i.e, chemical substances in their different dosage forms and strengths, we observed a near doubling in numbers from 132 to 243 products. Dermatologicals and alimentary tract and metabolism classes showed the highest variation (450 and 189%, respectively). In terms of dosage forms, around 20% of the purchases were injections and this rate varied by the therapeutic group. For example, they represented 13% of cardiovascular drugs and 50% of blood and blood-forming organs.

Finally, despite the increase in costs and drug mix, the volume of units purchased between 2012 and 2018 decreased by almost one third, from 1.7 billion to 1.2 billion units (Table 1). Overall, we found reductions in the purchases of 11 out of 13 classes and these reductions varied from -5% to -98%. The most substantial drop in volume was observed for genito-urinary drugs (98%), followed by dermatologicals (51%), and anti-parasitic products (44%).

**FIGURE 1:**
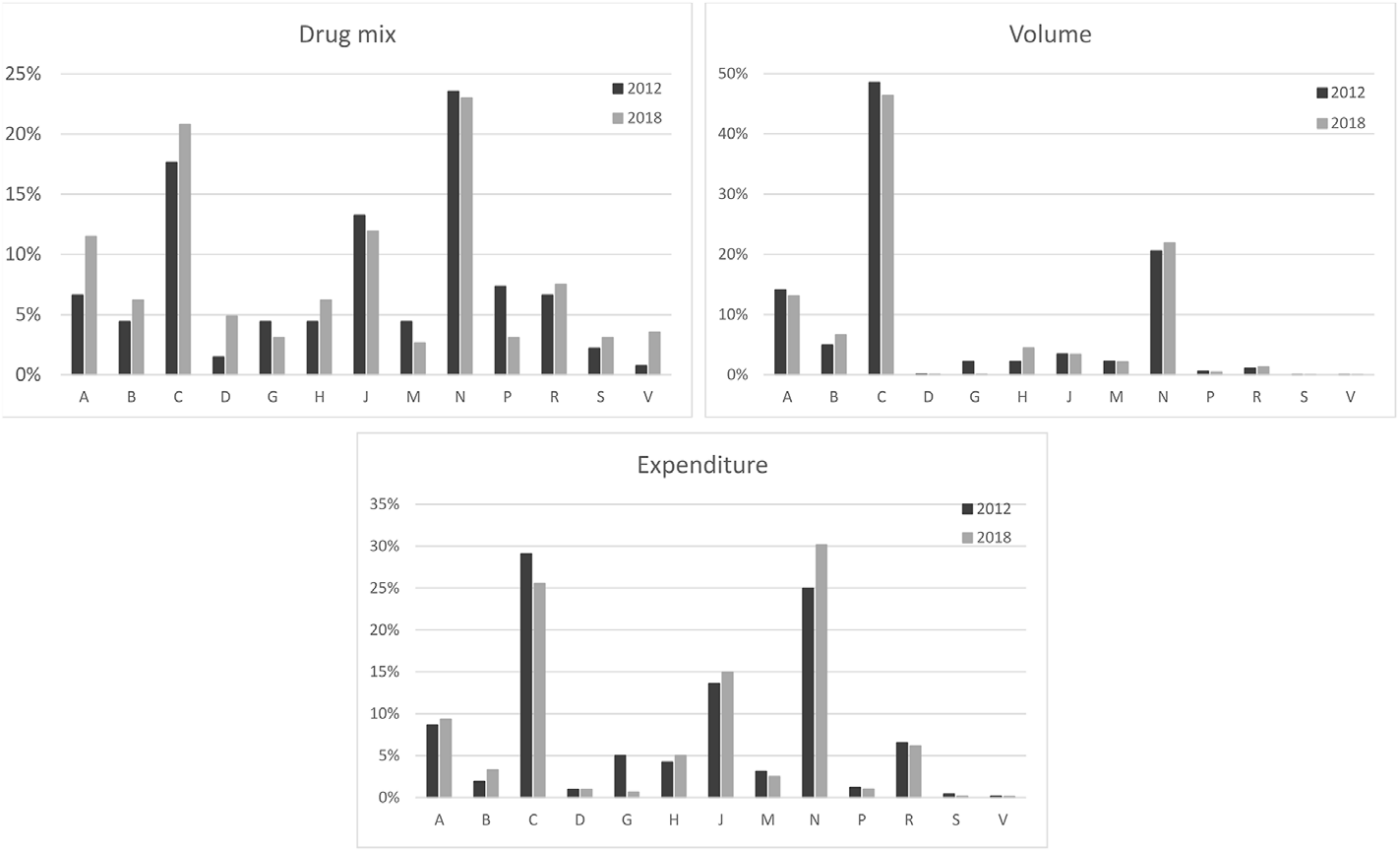
Distribution of the drug mix, volume, and expenditure by the major classes of medicines in 2012 and in 2018. Minas Gerais, Brazil.

Figure 1 shows the drug mix, volume, and expenditure indicators for the major classes of medicines for 2012 and 2018. As shown in the figure, purchasing was concentrated largely with a few classes of medicines. For instance, nervous system, cardiovascular, and systemic anti-infectives were responsible for more than half of the drug mix in 2012 and 2018. In terms of volume, cardiovascular, nervous system, and alimentary tract and metabolisms were the main classes (more than 80% of the total). We did find some difference in the classes for expenditures. In 2012, the cardiovascular, nervous system, and systemic anti-infectives were responsible for 67.7% of the total spending. By 2018, nervous system drugs changed position and led the spending, followed by cardiovascular, and systemic anti-infective medications. Together, these three major classes amounted to 70.6% of the financial resources in 2018.

### Decomposition analysis

The results of the decomposition analysis by major classes of medicines are presented in Table 2. Overall, the analysis suggests negative volume effects, while drug prices and drug mix had positive effects. In sum, this resulted in the increased expenditure we observed in 2018 compared to 2012. The expenditure index was positive for nine of the 13 major therapeutic classes, and the impacts largely arose from changes in prices and drug mixes. We observed positive volume effects for only two classes: systemic hormonal preparations and various (ATC V).

**TABLE 2:** Results of the decomposition analysis by the major classes of medicines. Minas Gerais, Brazil, 2012-2018.

Major classes of medicines^a^	Drug price effects (P)	Volume effects (V)	Drug-mix effects (D)	Expenditure index (E)^b^
A—alimentary tract and metabolism	1.37	0.65	1.39	1.23
B—blood and blood-forming organs	1.31	0.94	1.59	1.97
C—cardiovascular system	0.98	0.66	1.55	1.01
D—dermatologicals	1.13	0.49	2.65	1.47
G—genito-urinary system and sex hormones	0.22	0.02	38.69	0.15
H—systemic hormonal preparations, excl. Sex hormones and insulins	1.33	1.40	0.88	1.65
J—anti-infectives for systemic use	1.65	0.69	1.09	1.25
M—musculo-skeletal system	1.17	0.66	1.16	0.91
N—nervous system	1.36	0.74	1.31	1.32
P—anti-parasitic products, insecticides, and repellents	1.18	0.56	1.42	0.94
R—respiratory system	1.18	0.85	1.12	1.13
S—sensory organs	1.10	0.58	0.70	0.45
V—various	0.53	2.23	0.89	1.05
**TOTAL**	1.20	0.70	1.41	1.18

a ATC, first level.

b E = P x V x D = $\frac{\sum P_{1}V_{0}}{\sum P_{0}V_{0}}X\frac{\sum V_{1}}{\sum V_{0}}X\frac{\left(\sum P_{1}V_{1}/{\sum V}_{1}\right)}{\left(\sum P_{1}V_{0}/\sum V_{0}\right)}$

### Cardiovascular drugs

The overall findings for the cardiovascular class are presented in Table 3. Seven therapeutic subgroups of cardiovascular drugs were purchased in both 2012 and 2018. This corresponded to a volume of around 1.4 billion units and a total expenditure of US$17.8 million (BRL 69.0 million). While the mix of cardiovascular drugs doubled between 2012 and 2018, volumes dropped by 33% and expenditures remained similar.

**TABLE 3:** Cardiovascular and nervous system groups, drug mix, annual volume, and expenditure in 2012 and in 2018 and variations in the period. Minas Gerais, Brazil.

Therapeutic group^a^	Drug mix						Annual volume (millions)			Annual expenditure USD (millions)^b^		
	Chemical substance			Pharmaceutical products^c^								
	2012	2018	Variation^d^ (%)	2012	2018	Variation^d^ (%)	2012	2018	Variation^d^ (%)	2012	2018	Variation^d^ (%)
**C—Cardiovascular system**												
C01**—**Cardiac therapy	3	7	167	4	12	200	26.09	12.50	−52.11	0.51	0.77	52.78
C02**—**antihypertensives	2	3	50	3	5	67	22.48	15.46	−31.20	1.16	1.01	−13.10
C03**—**diuretics	3	3	0	3	5	67	185.68	106.13	−42.84	0.99	1.16	17.64
C07**—**beta blocking agents	4	4	0	5	12	140	133.95	98.61	−26.38	2.62	2.84	8.18
C08**—**calcium channel blockers	2	3	50	2	5	150	51.05	52.65	3.12	0.32	0.44	38.47
C09**—**agents acting on the renin–angiotensin system	3	3	0	5	5	0	353.29	193.20	−45.31	2.53	1.50	−40.48
C10**—**lipid modifying agents	1	1	0	2	3	50	44.05	64.95	47.44	0.73	1.18	60.77
**Total**	18	24	33	24	47	96	816.59	543.50	−33.44	8.85	8.90	0.56
**N—Nervous system**												
N01—anesthetics^e^	0	4	-	0	6	-	0	0.01	-	0	0.02	-
N02**—**analgesics	2	3	50	3	6	100	52.80	33.13	−37.25	0.86	0.97	12.33
N03**—**antiepileptics	5	5	0	9	12	33	113.48	87.79	−22.64	3.00	3.24	7.96
N04**—**anti-parkinson drugs	3	3	0	4	5	25	20.13	18.83	−6.49	0.73	2.25	207.24
N05**—**psycholeptics	4	5	25	10	14	40	78.49	40.05	−48.97	1.29	1.68	30.28
N06**—**psychoanaleptics	5	4	−20	6	9	50	81.21	76.99	−5.19	1.72	2.35	37.12
**Total**	19	24	26	32	52	63	346.11	256.81	−25.80	7.61	10.51	38.13

a ATC, second level.

b 1USD, 3.8742 BRL.

c Pharmaceutical products including all chemical substances in their different dosage forms and strengths.

d Variation = [(∑2018-∑2012)/∑2012]∗100.

e N01-anesthetics were only acquired in 2018.

In terms of specific therapeutic groups, beta blocking agents, agents acting on the renin–angiotensin system (RAS), and cardiac therapy accounted for almost 60% of the drug mix in 2012. In 2018, the drug mix was dominated by cardiac therapy and beta-blocking agents. In terms of volume, three groups were most relevant in this order: RAS-acting agents, diuretics, and beta-blocking agents. These groups corresponded to 80 and 73% of the volume purchased in 2012 and 2018, respectively. In terms of expenditures, beta-blocking agents, RAS-acting agents, and anti-hypertensives, in this order, accounted for 71.2% of the total (USD 6.31 million) in 2012. In 2018, lipid-modifying agents replaced anti-hypertensives for third position.

The decomposition analysis results for cardiovascular medications are presented in Table 4. We found that expenditures increased for five therapeutic subgroups, while they decreased for two. Of the underlying factors driving these changes, drug mix had positive effects for all the seven subgroups, while price had positive effects for four. Volume increased for only lipid-modifying agents and calcium channel blockers. The most pronounced positive effect of price (*p* = 2.77) and negative effect of volume (V = 0.48) for the expenditure index was observed for cardiac therapy drugs, while drug mix had the strongest effect on anti-hypertensives (D = 2.16).

**TABLE 4:** Results of the decomposition analysis by cardiovascular and nervous system groups. Minas Gerais, Brazil, 2012–2018.

Therapeutic group^a^	Drug price effects (P)	Volume effects (V)	Drug-mix effects (D)	Expenditure index (E)^b^
C—cardiovascular system				
C01—cardiac therapy	2.77	0.48	1.15	1.52
C02—anti-hypertensives	0.60	0.55	2.16	0.72
C03—diuretics	1.42	0.57	1.45	1.17
C07—beta-blocking agents	0.75	0.74	1.97	1.08
C08—calcium channel blockers	1.26	1.03	1.07	1.39
C09—agents acting on the renin–angiotensin system	0.82	0.55	1.34	0.60
C10—lipid-modifying agents	1.01	1.47	1.08	1.60
Total	0.98	0.66	1.55	1.01
**N**—**Nervous system** ^**c**^				
N02—analgesics	1.85	0.63	0.97	1.12
N03—antiepileptics	1.01	0.77	1.42	1.12
N04—anti-parkinson drugs	0.82	0.93	3.49	2.69
N05—psycholeptics	1.16	0.51	1.55	0.92
N06—psychoanaleptics	1.39	0.95	1.06	1.40
Total	1.36	0.74	1.31	1.32

a ATC, second level.

^b^ E = P x V x D = $\frac{\sum P_{1}V_{0}}{\sum P_{0}V_{0}}X\frac{\sum V_{1}}{\sum V_{0}}X\frac{\left(\sum P_{1}V_{1}/{\sum V}_{1}\right)}{\left(\sum P_{1}V_{0}/\sum V_{0}\right)}$

c N01-anesthetics were only acquired in 2018 and because of that there is no decomposition analyses for this group.

### Nervous system drugs

Table 3 also shows the breakdown of therapeutic groups for nervous system drugs, where six different therapeutic groups were purchased. These purchases were for a volume of 603 million pharmaceutical units and a total expenditure of USD 18.1 million (BRL 70.1 million). Anti-epileptics, psychoanaleptics, and psycholeptics were responsible for nearly 80% of both the total volume purchased and total expenditures. These drugs also accounted for 80% of the drug mix, with a slightly different ordering: psycholeptics, psychoanaleptics, and anti-epileptics, respectively.

Across the subgroups of nervous system drugs, our decomposition analysis showed that the expenditure index was positive for four subgroups and negative for one subgroup (Table 4). Prices also increased for the four subgroups (analgesics, anti-epileptics, psycholeptics, and psychoanaleptics), and this increase was most pronounced for analgesics (*p* = 1.85). At the same time, volumes decreased across all of the subgroups. Psycholeptics had the greatest reduction in volume (V = 0.51). Changes in the drug mix also had positive effects on the expenditures for four subgroups, particularly anti-Parkinson drugs (D = 3.49).

## Discussion

In general, many pharmaceutical policies are intended to improve the provision and use of medicines (Morrow, 2015). The ERAF policy was launched as a state-level policy to improve medicine procurement within the PCHPS program in Minas Gerais. This policy established a new decentralized procurement system, in which decision making, responsibility, and control was moved from a centralized system to municipal managers starting in 2016 (SES/MG - Secretaria de Estado de Saúde de Minas Gerais, 2016). This decentralization in the public procurement process is not new and is supported by the theory that local managers are more familiar with their programmatic needs, and thus can increase the efficiency of purchasing processes (McCue et al., 2000). It was hoped that the policy would lead to the provision of quality medicines at public facilities, in the right quantities, at the right time (Fausto et al., 2017, Paim et al., 2011, Brazil, 2006a, b).

Our evaluation focused on the operational goals of the policy: price reductions, volume increases, and drug mix expansions. Overall, medicine expenditure increased by 14.5% after ERAF implementation while the quantities purchased decreased 30%. The only indicator showing an alignment with the policy goals was the drug mix.

It is important to note, that just the three therapeutic classes—nervous system, cardiovascular, and systemic anti-infectives—accounted for 70% of the total financial resources invested by the municipalities. This suggests a discrepancy between purchasing and the PHCPS’ goal of covering treatments for various conditions. Notably, this concentration leaves comparatively little spending for major causes of diseases, including diabetes mellitus, chronic respiratory conditions, parasitic diseases, iron-deficiency anemia, and gynecological conditions (GBD 2016 Brazil Collaborators, 2018). Importantly, several of these diseases with less expenditure focus are among the leading non-communicable causes of mortality in Brazilian municipalities (Cardoso et al., 2021), creating inequity in access to medicines. Our results are in line with a nationwide survey with a representative sample of 12,725 adults aged 20 years or more, which have shown that free access to cardiovascular treatments was favored, while those medicines acting on the respiratory system were more often paid out-of-pocket (Tavares et al., 2016).

One of the main arguments favoring the implementation of the ERAF policy was to enhance the quantification process, avoiding risks of shortages of specific treatments and wastages, problems pointed out by a report from the State Audit Court in 2013 (TCE/MG - Tribunal de Contas do Estado de Minas Gerais, 2013). Indeed, several studies have highlighted that about half of the essential medicines were available at PHCPS in Minas Gerais (Barbosa et al., 2021, 2017; Luz et al., 2017a; Lima et al., 2019). Our findings, however, do not support the rationale that decentralization of quantification to municipalities would improve the process since there was a significant drop in volume associated with the investments in few therapeutic classes. Instead, our findings corroborate the studies based on data collected after ERAF implementation showing that low availability of medicines has continued to impact medicine access to primary care patients (Nascimento et al., 2020; Bueno et al., 2021).

Considering that the ERAF policy was established to better coordinate procurement in an effort to reduce costs, our findings do not support its success in that regard. The decomposition analysis confirmed a spending growth in 2018 in comparison with 2012. Expenditure drivers were the increase-in-the-price component by 20% and in the drug mix by 41%, whereas the quantity component decreased by 30%. The same behavior for each therapeutic class was observed individually: positive effects of the price and of the drug mix, and negative effects of volume. These findings indicate that, from 2012 to 2018 there was a shift toward more expensive products and higher prices were paid for the existing ones. This likely contributed to the lower volumes purchased, considering the annual cap on the PHCPS budget (Brazil, 2017b).

Medicines costs are rising faster worldwide. Although Brazil implemented pharmaceutical policies for more than 20 years (Brazil, 2004,^1998^) on top of a large body of legislation and guidelines around public procurement of medicines, transparency, accountability, and functioning of the PHCPS (Brazil, 2012, 2017a, 2017c; Kohler et al., 2015; Rida et al., 2017; Bermudez et al., 2018), the evidence does not support their effectiveness in reducing prices and increasing medicine availability (Brazil, 1998; Kohler et al., 2015; Tavares et al., 2016; Nascimento et al., 2017; Bueno et al., 2021).

Lack of governance, insufficient monitoring, poor implementation of regulations, along with a restricted number of producers and suppliers in the pharmaceutical market are possible reasons for the failure of the pharmaceutical pricing policies, especially in developing countries (Kohler et al., 2015; Rida et al., 2017). Additionally, pricing interventions in healthcare systems where the government procures medicines are limited to applying competitive bidding for a restricted list of medicines. Furthermore, robust generic substitution policies are not usually in place, neither are policies focusing on innovation and strategies to stimulate the local pharmaceutical industry (Gray, 2015). It is reasonable to assume that a combination of these factors influences the price increase observed in our study. Drug prices may also have increased due to the ERAF requirement that states that deliveries are to be made to municipalities rather than to the state’s central warehouse.

The observed increase in drug mix likely resulted from the ERAF-initiated change to municipalities following the Brazilian national list of essential medicines–RENAME–as the reference list (SES/MG - Secretaria de Estado de Saúde de Minas Gerais, 2016), which is more comprehensive than the prior municipal lists (Assunção et al., 2013). Even here, however, the observed rise of the drug mix was uneven across therapeutic classes and resulted more from the number of pharmaceutical products rather than chemical substances.

Our further analyses allowed us to enhance our understanding about the spending patterns on cardiovascular and nervous system drugs, groups that accounted for 55% of the expenditure both in 2012 and in 2018.

The cardiovascular group includes anti-hypertensives, cardiac therapy, beta-blocking agents, RAS-acting agents among others, while the nervous system group contains, for example, analgesics, anxiolytics, antidepressants, hypnotics, and sedative drugs. Thus, the relevance of these groups among all the therapeutic groups procured was, at a certain degree, predictable, since they incorporate drugs to treat a wide variety of prevalent illnesses and acute conditions. In Minas Gerais, for instance, a cross-sectional study with a representative sample of primary care patients aged 18 years or older in 104 municipalities found that hypertension (50.3%), dyslipidemia (31.2%), depression (28.0%), and arthritis/arthrosis or rheumatism (20.4%), in this order, were the most prevalent diseases (Moreira et al., 2020).

Although the expenditure on the cardiovascular group remained the same in 2018 compared to 2012, of particular note is the significant decrease in the volume and expansion of the drug mix. Noteworthily, the growth in the drug mix was due to changes not in terms of incorporation of novel cardiovascular therapies, but regarding pharmaceutical products, i.e., it was purchasing the same drugs in different dosage forms and strengths. This was particularly relevant for beta-blocking agents, drugs acting on the renin–angiotensin system (RAS), lipid-modifying agents, and diuretics. For example, almost 50% of the spending was centralized on beta-blocking agents and RAS-acting agents, and the main changes on the drug mix in 2018 were the purchase of products such as carvedilol tablets (6.25 and 25 mg), metoprolol tablets (25 and 100 mg), and propranolol tablet 10 mg among the beta-blocking agents, and enalapril tablet 5 mg among the RAS drugs.

The marked reduction in the volume of cardiovascular drugs purchased may significantly compromise millions of patients’ well-being and quality of life. Taking hypertension as an example and considering the risks of heart, brain, and kidney diseases (Hevesi et al., 2018; Volpe and Savoia, 2018) that accompany patients if untreated, the Brazilian guideline recommends anti-hypertensives, diuretics, beta-blocking agents, calcium channel blockers, and RAS-acting agents for the treatment (Brazil, 2013). Our findings highlighted that, except for calcium channel blockers which had a quite small increase in the volume in 2018 compared to 2012, all the other groups had lessened their quantities by figures ranging from 26 to 45%. These results are consistent with (Mengue et al. (2016)), who found that only 56% of patients could access their anti-hypertensive treatment through the National Health Care System (SUS).

With respect to nervous system drugs, the expenditure increased by 40% and this finding contrasts with our previous study on neuropsychiatric drug procurement in Minas Gerais (Carvalho et al., 2021). Unlike the investigation that included several institutions and purchases made by the Minas Gerais state-level management, this study focused only on medicines procured within the PHCPS program, which may explain the difference in the results.

The expenditure drivers were price and drug mix increases (36 and 31%, respectively) and volume decrease (26%), which means that the municipalities are paying more for less and choosing products that are, on average, more expensive. As in cardiovascular drugs, the mix did not expand due to the inclusion of new therapies, but mostly because of purchasing different dosage forms and strengths for the existing drugs. This was the case, for instance, of anti-epileptics (e.g., addition of phenytoin 50 ml/ml injection, phenobarbital 100 mg/ml injection, sodium valproate 500 mg tablet, and carbamazepine 400 mg tablet); psycholeptics (e.g. chlorpromazine 5 mg/ml injection, diazepam 5 mg/ml injection, and haloperidol 1 mg tablet); and psychoanaleptics (e.g., amitriptyline 75 mg tablet, clomipramine 10 mg tablet, nortriptyline 75 mg capsule, and nortriptyline 10 mg capsule).

Anti-Parkinson drugs presented the highest expenditure variation (207%) and the most significant effect on expenditure was caused by the drug mix (increase by a factor of 3.5). The findings on anti-Parkinson drugs are also consistent with the results from our previous study (Carvalho et al., 2021), where we observed an upward trend in pharmaceutical spending on these drugs. In that study, the drug mix also played a leading role in the expenditure, although not exclusively (in that case, the volume was the strongest factor).

The changes in the drug mix we observed in this investigation were due to pharmaceutical products (e.g., incorporation of biperiden 5 mg/ml injection) and also to the purchase of greater volumes of more expensive products, such as levodopa 200 mg plus benzerazide 50 mg, again, another common finding with our previous study (Carvalho et al., 2021). These products were responsible for 20% of the volume purchased in 2018. Although biperiden injection is classified for primary care use at the RENAME (Brazil, 2018b), this product is not recommended at the Brazilian national protocol for Parkinson disease (PD) (Brazil, 2017d), which sets the biperiden tablet (2 and 4 mg) for the initial treatment phase of the disease. Levodopa in combination with benzerazide is at RENAME and at the official guideline, which supports its effectiveness in the symptomatic control of PD (Brazil, 2017d).

### Strengths and limitations

To the best of our knowledge, this is the first study examining the performance of the ERAF policy considering its goals of price reduction, volume increase, and drug mix expansion through the PCHPS program. Some limitations, however, deserve consideration. We opted to measure volumes in units of the drugs purchased instead of DDDs because several drugs registered in the SIGAF database were not classified in the ATC-DDD system, thus limiting some comparisons. Although we outlined several pharmaceutical products to discuss the study findings, our unity of analysis was the aggregated therapeutic classes purchased, not the individual drugs. Furthermore, we could only estimate direct drug expenditures (volume x price) and no other costs to the healthcare system, for instance, such as the costs associated with distribution and storage. Nevertheless, our study relied on comprehensive data from SIGAF from almost every municipality in Minas Gerais (only 5.8% of non-differentiated losses), meaning our external validity and generalizability are likely very high.

### Implications for policy and practice

The evidence provided by this study suggests some avenues to develop a more sustainable long-term policy in this area. First, the adoption of the Brazilian national medicines list–RENAME–could be reconsidered since this list, from 2012 on, has changed its purpose from indicating essential medicines to specifying medicines financed by the public system (Osorio-de-Castro et al., 2018). Second, the option for a more limited number of drugs selected should lead to a better supply at lower costs (Management Sciences for Health, 2012). Third, employing evidence-based sources for medicine selection, such as scientific literature and national and international therapeutic guidelines and protocols could also support the process (Management Sciences for Health, 2012). Finally, to reduce the risk of treatment non-adherence, it would be preferable to select, whenever possible, oral pharmaceutical formulation instead of injections, as they are more convenient and preferred routes of administration by patients (Stewart et al., 2016).

Investments could be made, by the municipalities, to improve the quantification process, which should be combined with monitoring and periodic reviews by state-level managers and policymakers (Management Sciences for Health, 2012).

Regarding prices, our results suggest that more attention should be paid to the bidding processes, and policies focusing on increasing the value for money (UK-Government, 2020), i.e., providing medicines of appropriate quality and effectiveness at the least possible cost. Contracting authorities in the municipalities should ensure regular feedback to state managers about the suppliers’ performance, creating a monitoring network to exclude those that do not have the capability to deliver quality products on time.

## Conclusions

This study evaluated the performance of a new and unique pharmaceutical policy in Brazil, implemented by one of its most populous states. Overall, our findings suggest that the intervention proposed by the Minas Gerais Government–the ERAF policy–cannot be considered effective regarding its outputs, as it has not made an empirical contribution to the achievement of the intended purposes so far (OECD - Organisation for Economic Co-operation and Development, 2009).

Running a public healthcare system of this magnitude is challenging, especially considering the goal of supplying medicines free of charge to the population. The ERAF policy can be revised to deal with the drawbacks we identified to maximize procurement practices. The experiences and lessons learned in Minas Gerais are useful for countries where health systems aim to allow appropriate and sustainable access to medicines to their populations.

## Data Availability

The dataset supporting this article can be made available upon request after approval by the corresponding author.
